# Editorial: Environmental challenges and endocrine dysregulation

**DOI:** 10.3389/fendo.2025.1717971

**Published:** 2025-10-21

**Authors:** Cuiqing Liu, Yanhong Wei, Maaike Kockx, Leonard Kritharides, Kezhong Zhang

**Affiliations:** ^1^ School of Public Health, Zhejiang Chinese Medical University, Hangzhou, China; ^2^ Department of Toxicology, School of Public Health, Sun Yat-sen University, Guangzhou, China; ^3^ ANZAC Research Institute, Concord Repatriation General Hospital, Concord, NSW, Australia; ^4^ Department of Cardiology Concord Repatriation General Hospital, Concord Clinical School, Faculty of Medicine and Health, University of Sydney, Concord, NSW, Australia; ^5^ Center for Molecular Medicine and Genetics, Wayne State University School of Medicine, Detroit, MI, United States; ^6^ Department of Biochemistry, Microbiology, and Immunology, Wayne State University School of Medicine, Detroit, MI, United States

**Keywords:** environmental challenges, endocrine dysregulation, PM_2.5_, metabolic disorders, public health

The growing burden of metabolic and endocrine disorders in the global population has prompted research efforts on the multifactorial origins of these diseases. Over the past decade, accumulating evidence from epidemiological, clinical, and experimental studies has illustrated the profound impact of environmental exposures on hormonal regulation, energy metabolism, and cardiometabolic outcomes. Environmental stressors, including fine airborne particulate matters PM_2.5_, endocrine-disrupting chemicals, and climate-related exposures, have emerged as critical drivers of metabolic dysregulation and endocrine pathology (1, 2). This Research Topic, *Environmental Stressors and Metabolic Disease*, provides some insights into how air pollutants, heavy metals, synthetic chemicals, and even naturally occurring compounds such as terpenes interact with genetic, physiological, and social factors to modulate endocrine function and disease risk.

The review article by Cocchi et al. examined how environmental pollution, socioeconomic disparities, climate stressors, and life-course transitions such as menopause and pregnancy collectively increase cardiovascular disease (CVD) risk in women with type 2 diabetes mellitus (T2DM). The review integrates data across reproductive stages, from gestational diabetes to menopause, to demonstrate how environmental pollutants, such as PM_2_._5_ and ozone, climate-induced food insecurity, and chronic psychosocial stressors synergistically exacerbate insulin resistance, systemic inflammation, and endothelial dysfunction. The authors argue for sex-specific, life stage-adapted public health frameworks that reflect the cumulative impact of environmental and social exposures on female cardiometabolic health.

An additional review article by Wright et al. presents a mechanistic summary on the endocrine-disrupting properties of atrazine (ATZ), a widely used herbicide linked to breast cancer development. The authors explore the potential of curcumin, a phytochemical found in turmeric, as a promising therapeutic agent capable of attenuating ATZ-induced dysregulation in estrogen signaling and proliferation pathways. They propose curcumin as a potential detoxifying agent, capable of modulating multiple molecular targets, including CYP3A4 induction, CYP19A1 suppression via miR-125a and ERRα, and inhibition of the EGF signaling cascade. By bridging molecular toxicology and phytopharmacology, this review highlights the potential of natural compounds in mitigating environmental toxicity.

The research article contributed by Wen et al. offers epidemiological evidence linking occupational exposure to multiple metals, particularly iron and copper, with a high prevalence of thyroid nodules in oilfield workers. Using both logistic and Weighted Quantile Sum (WQS) regression models, the study supports the hypothesis that chronic occupational exposure to metal mixtures may dysregulate thyroid homeostasis, possibly via oxidative stress and trace metal-mediated modulation of thyroid hormone synthesis. This study emphasizes the growing health concerns in industrial populations exposed to complex mixtures of environmental metals and supports the use of mixture models such as WQS regression to understand these interactions.

Further expanding the scope of environmental toxicants, Nie et al. investigated the relationship between terpene exposure and metabolic syndrome using data from National Health and Nutrition Examination Survey (NHANES). Their studies revealed significant associations, particularly with limonene and pinene, mediated by inflammatory pathways. The authors apply advanced statistical techniques including Bayesian Kernel Machine Regression (BKMR) and Quantile g-Computation and identify systemic inflammation as a potential mediator. The findings raise concerns about indoor air quality and highlight an emerging class of metabolic disruptors warranting regulatory oversight.

In a preclinical study, Chen et al. demonstrated that dapagliflozin, a sodium-glucose cotransporter 2 inhibitor (SGLT2i), improves insulin sensitivity, hypothalamic inflammation, and fertility markers in female mice subjected to chronic high-fat diet. This study reveals that dapagliflozin improves fertility and hormone regulation, even when systemic inflammation is not significantly altered, suggesting its potential in treating environmentally or diet-induced ovulatory disorders.

Finally, a review article by Saleem et al. summarizes toxicological evidence linking smog exposure to disruption of multiple endocrine axes. Smog is a form of air pollution comprising of gases, such as ozone, sulfur dioxide, nitrogen and carbon oxides, and solid particulate matters, PM_2.5_ and PM_10_. The article links oxidative stress, epigenetic modification, and hormone receptor interference with a wide range of disorders, including insulin resistance, infertility, and endocrine cancers.

Together, the articles in this Research Topic illustrate the multifaceted ways in which environmental stressors contribute to endocrine dysfunction and metabolic disease. From air pollution and industrial chemicals to social inequality and climate change, the external environment increasingly emerges as a major player in the development and progression of endocrine disorders at the modern time (3, 4). As the global burden of metabolic diseases continues to rise, interdisciplinary research that bridges environmental science, endocrinology, and public health is not only warranted, but also essential. We thank the contributing authors for their informative work and the reviewers for their insightful feedback. We hope this Research Topic serves as a catalyst for future efforts on the complex interplay between environment and endocrine health.

## References

[1] Zhang K. Environmental PM(2.5)-triggered stress responses in digestive diseases. eGastroenterology. (2024) 2:e100063. doi: 10.1136/egastro-2024-100063, PMID: 38895535 PMC11185827

[2] Ge Q Yang S Qian Y Chen J Yuan W Li S. Ambient PM2.5 exposure and bone homeostasis: analysis of UK biobank data and experimental studies in mice and *in vitro* . Environ Health Perspect. (2023) 131:107002. doi: 10.1289/EHP11646, PMID: 37792558 PMC10549986

[3] Hou T Jiang Y Zhang J Hu R Li S Fan W. Kidney injury evoked by fine particulate matter: risk factor, causation, mechanism and intervention study. Adv Sci (Weinh). (2024) 11:e2403222. doi: 10.1002/advs.202403222, PMID: 39316383 PMC11578332

[4] Zhang K. Environmental diseases. Environ Dis. (2016) 1:1–2. doi: 10.4103/2468-5690.180330 PMC5336312 28265594

